# Herpes simplex virus alkaline nuclease interacts directly with ubiquitin-specific protease 15 through a conserved C-terminal domain

**DOI:** 10.1016/j.jbc.2026.111444

**Published:** 2026-04-09

**Authors:** Alexander Leehangin, Tyler D. Hasselmann, Jessica Pagelkopf, Rebecca Hamel, Patricia Devaux, Kareem N. Mohni

**Affiliations:** Department of Molecular Medicine, Mayo Clinic, Rochester, Minnesota, USA

**Keywords:** herpesvirus, ubiquitin-dependent protease, protein–protein interaction, protein domain, DNA enzyme, nuclease

## Abstract

Herpes simplex virus replicates in the nucleus of the host cell and utilizes many cellular proteins to facilitate DNA replication. The deubiquitinating enzyme ubiquitin-specific protease 15 (USP15) is one of the most enriched proteins on replicating viral DNA and is recruited to the nucleus by a direct interaction with the viral alkaline nuclease, UL12. To better define this protein–protein interaction, we applied genetic and structural approaches and have mapped the USP15 binding domain to three conserved amino acids on the UL12 C-terminal alpha helix. A small internal deletion in this helix disrupts the UL12 interaction with USP15 but not other UL12-interacting proteins. Bioinformatic analysis reveals that this alpha helix is strongly conserved in all alphaherpesviruses but not in betaherpesviruses or gammaherpesviruses. The Kaposi's sarcoma–associated herpesvirus homolog, SOX, has an alpha helix in the same position, but the amino acid composition is highly divergent compared with UL12. Furthermore, overlaying the HerpesFolds predictions of UL12 with SOX shows high structural similarity in the nuclease domains but marked divergence in the C-terminal helix. We demonstrate that the UL12 C-terminal alpha helix can be swapped with another alphaherpesvirus UL12 helix and maintain the ability to recruit USP15 to the nucleus. Conversely, substitution with the divergent SOX helix maintains a folded protein with nuclease activity but fails to recruit USP15 to the nucleus. Analysis of the sequence between the alphaherpesvirus UL12 helices identified six identical residues. We functionally validated three of these amino acids as required for USP15 interaction. These observations identify the USP15 binding domain on UL12.

The Orthoherpesviridae family includes all nine human herpesviruses (1). Viruses in this family have large, linear, double-stranded DNA genomes that replicate in the nucleus of the host cell. Following primary infection, they all establish latency and can be periodically reactivated during the life of the host. This family can be further subdivided into three subfamilies consisting of Alphaherpesvirinae (alpha), Betaherpesvirinae (beta), and Gammaherpesvirinae (gamma) (1). Human alphaherpesviruses include herpes simplex viruses 1 and 2 (HSV-1/2) as well as varicella zoster virus; betaherpesviruses include human cytomegalovirus (HCMV) and human herpesviruses, HHV-6A, HHV-6B, and HHV-7, and gammaherpesviruses include Epstein–Barr virus (EBV) and Kaposi's sarcoma–associated herpesvirus (KSHV). Approximately 60% to 90% of the world population is infected with HSV-1 (2).

All members of the Orthoherpesviridae replicate their genomes by the formation of concatemers, longer-than-unit-length molecules that consist of multiple copies of head-to-tail arrangements of the viral genome (3, 4, 5). The formation of concatemers is an evolutionarily conserved mechanism extending all the way to bacteriophages (6). Concatemer formation is thought to be facilitated by a 5′ to 3′ exonuclease and a single-strand annealing protein, typified by bacteriophage λ Redα/β (7, 8). HSV-1 encodes an analogous two-component system, including UL12, the 5′ to 3′ alkaline exonuclease, and ICP8, the single-strand annealing protein (9, 10, 11, 12).

Lytic infection with HSV-1 results in a dramatic reorganization of the host cell nucleus and the formation of large replication compartments. Replication compartments are nucleated by ICP8 and represent the main sites of viral RNA transcription, DNA replication, and genome packaging (13, 14, 15, 16). ICP8 itself interacts with a large number of host DNA replication and repair proteins (17). Furthermore, the initiation of viral DNA replication elicits a cellular DNA damage response characterized by autophosphorylation of the ATM kinase and the phosphorylation of many of its downstream substrates, including Nbs1, CHK2, and 53BP1 (18, 19, 20). This activation of ATM is thought to facilitate viral DNA replication and production of infectious viruses (19, 21).

HSV-1 UL12 is a processive 5′ to 3′ exonuclease with a preference for high pH and a divalent metal cation but not ATP (22, 23, 24). The enzyme also exhibits minor endonuclease activity (22, 25, 26). The exonuclease activity of UL12 is essential for the production of infectious virus (11, 27). Viral DNA replicated in the absence of UL12 accumulates complex structures that are unable to enter a pulsed-field gel, even after digestion with restriction endonucleases (28, 29). UL12 is also sufficient to stimulate homology-directed repair of DNA double-strand breaks by a single strand–annealing mechanism (10, 30). The most studied UL12 homologs include UL98 (HCMV), BGLF5 (EBV), and SOX (KSHV), which all contain the conserved enzymatic core and are required for optimal virus growth (31, 32, 33, 34).

UL12 is expressed early during infection and is recruited to viral replication compartments (21). UL12 also physically interacts directly with several DNA repair protein complexes, such as the double-strand DNA break sensor MRN, consisting of the Mre11, Nbs1, and Rad50 subunits, as well as the mismatch repair heterodimers MSH2/3 and MSH2/6 (21, 35). We have recently identified a new UL12-interacting protein, ubiquitin-specific protease 15 (USP15) (30). UL12 interacts directly with USP15 and physically recruits it to the nucleus of infected cells to promote viral DNA replication and recombination. USP15 also functions to regulate the stability of UL12 in both infected cells and cells transfected with a UL12 expression plasmid (30). In this study, we utilize genetic and biochemical data to identify and characterize a functional USP15-interacting domain on UL12 that is conserved across the alphaherpesviruses but not betaherpesviruses or gammaherpesviruses.

## Results

### The C terminus of UL12 contains the USP15 binding domain

**Figure 1:**
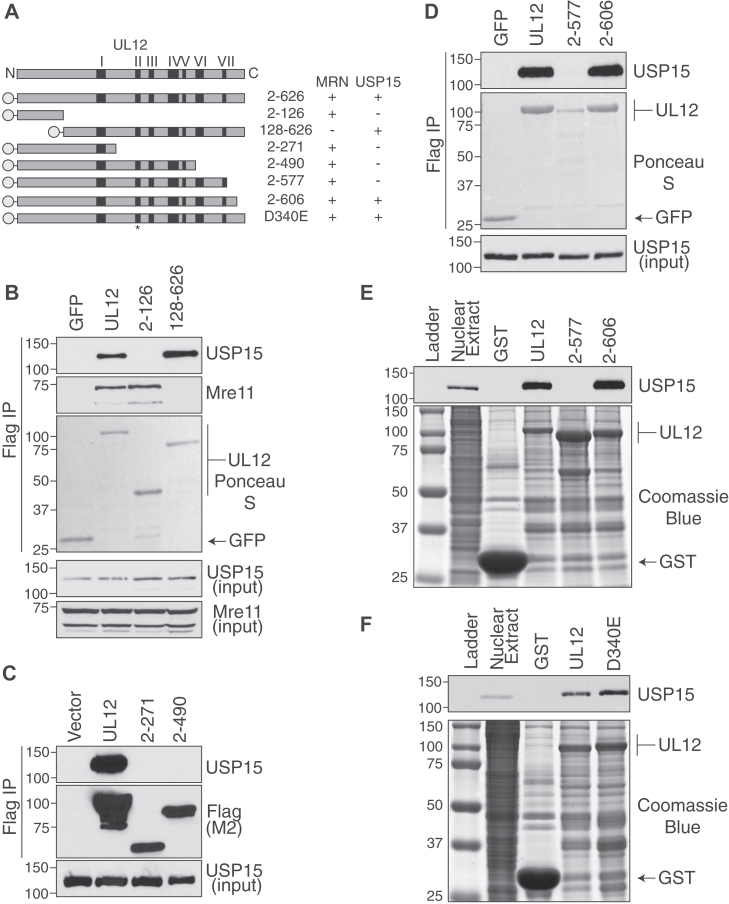
**USP15 binding region is located between amino acids 577 and 606 on UL12.***A,* schematic and summary of the results for the various truncations and point mutants tested. The *black bars* indicate the seven conserved nuclease motifs present in alkaline nucleases. The *N-terminal circles* represent the tag added for purification. For experiments in mammalian cells, UL12 was expressed with an N-terminal GFP-FLAG-NLS, and for experiments in bacteria, UL12 was expressed with an N-terminal GST. The figure is drawn to scale. *B*–*D,* the indicated UL12 truncation mutants were expressed in 293T cells, immunoprecipitated with FLAG beads, and immunoblotted for USP15 as indicated. UL12 was visualized either by Ponceau S staining of the membrane or by immunoblot for FLAG. *Arrows* indicate the respective sizes of GFP and UL12. *B,* UL12, 2 to 126, and 128 to 626. *C,* UL12, 2 to 271, and 2 to 490. *D,* UL12, 2 to 577, and 2 to 606. *E* and *F,* the indicated UL12 truncations or point mutants were expressed in Arctic Express bacteria and purified on glutathione sepharose resin. After extensive washing, UL12-bound resin was incubated in 293T nuclear extracts. UL12 was visualized by Coomassie blue staining of the gel. *Arrows* indicate the respective sizes of GST and UL12. USP15 copurifying with UL12 was identified by immunoblot. *E,* UL12, 2 to 577, and 2 to 606. *F,* UL12 and exonuclease-deficient mutant D340E. GST, glutathione-S-transferase; USP15, ubiquitin-specific protease 15.

We have previously reported that the HSV-1 alkaline nuclease, UL12, interacts directly with cellular USP15 (30). The previously described interaction between UL12 and the cellular MRN complex maps to the N-terminal unstructured region of UL12 (21). To determine if USP15 and MRN share the same binding mechanism with UL12, we tested whether USP15 can also interact with the N terminus of UL12. FLAG-tagged full-length UL12 (amino acids 2–626), 2 to 126, and 128 to 626 were expressed in 293T cells and immunoprecipitated with FLAG antibodies. Mre11 copurified with full-length UL12 and 2 to 126, but not with 128 to 626 (Fig. 1, *A* and *B*), as previously published (21). USP15 copurified with full-length UL12 and 128 to 626 but not 2 to 126 (Fig. 1, *A* and *B*), indicating that USP15 interacts with the C terminus of UL12 and does not share the same binding site as the MRN complex. To further define the region of UL12 that interacts with USP15, we utilized two C-terminal truncations that were previously described and maintain the interaction with MRN (21). Neither 2 to 271 nor 2 to 490 interacted with USP15 by coimmunoprecipitation (Fig. 1, *A* and *C*), indicating that the USP15 binding domain is C terminal to amino acid 490 on UL12.

Genetic data combined with recent structural modeling of UL12 revealed that UL12 contains a conserved and structured nuclease domain that ranges from amino acids 128 to 606 (11, 27). This domain consists of seven nuclease motifs that end at amino acid 576. The nuclease domain is flanked by the unstructured N-terminal domain, amino acids 2 to 126, as well as a short unstructured C terminus, amino acids 607 to 626 (6, 27, 36, 37). To further define the USP15 binding domain, we generated UL12 C-terminal truncations 2 to 606, which removes the unstructured C-terminal domain, as well as 2 to 577, which stops immediately after the final nuclease motif and deletes the final C-terminal alpha helix of the structured region. UL12 C-terminal truncation 2 to 606 maintained the interaction with USP15 by coimmunoprecipitation, whereas 2 to 577 lost the interaction (Fig. 1, *A* and *D*). Together, these data indicate that the USP15 binding domain is between amino acid residues 577 and 606 on UL12 and that the C-terminal unstructured domain is not required for the interaction.

During the analysis of these truncation mutants, we observed that the 2 to 577 truncation was expressed at lower levels than wildtype UL12 and 2 to 606 (Fig. 1*D*). There was also a significant laddering of the 2 to 577 protein present on the membrane, consistent with proteolytic degradation. We previously published that USP15 is required to maintain the stability of UL12 in both transfection experiments and during infection with wildtype virus (30). Based on these two observations, we hypothesized that 2 to 577 is unstable because it cannot interact with USP15, and the smaller bands represent biologically relevant degradation products. To confirm that USP15 was not detected because of the low levels of 2 to 577 purified, we employed a bacterial expression system for UL12. Previous attempts to purify UL12 from bacteria have resulted in insoluble protein, which needs to be refolded prior to further analysis (24). Here, we utilized Arctic Express BL21 cells, which contain a cold-adapted chaperone protein, allowing for low-temperature induction and proper folding of the protein of interest. A glutathione-*S*-transferase (GST)-UL12 fusion protein induced with this system expressed to high levels and remained in the soluble fraction following cell lysis (Fig. 1*E*). GST, GST-UL12, and GST-C-terminal truncations were induced at low temperatures, and soluble lysates were incubated with glutathione sepharose resin. Protein-bound resin was then incubated with 293T nuclear extracts, and copurifying USP15 was identified by immunoblotting. USP15 was efficiently pulled down with UL12 and 2 to 606, but not with 2–577, despite the higher expression levels of this truncation in the bacterial system (Fig. 1*E*). This indicates that 2 to 577 does not interact with USP15, and the observation is not an artifact of purifying less protein from the mammalian cell culture system. Finally, we also induced exonuclease-dead full-length UL12 D340E and demonstrated that it maintained the interaction with USP15 (Fig. 1*F*), indicating that the exonuclease activity of UL12 is not required for the interaction.

### The USP15-interacting domain on UL12 consists of conserved amino acids 584 to 604

**Figure 2:**
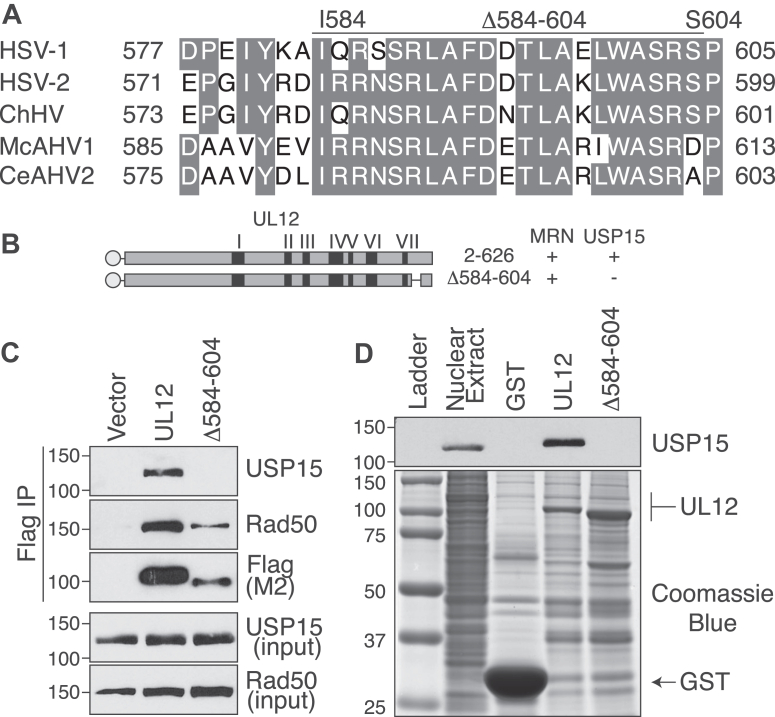
**USP15 binding region is located in conserved amino acids between 584 and 604 on UL12.***A,* amino acid sequence alignment of HSV-1 USP15 binding region 577 to 605 on UL12 with other human and primate alpha herpes simplex viruses. Identical amino acids are highlighted in *gray*. The *bar* on *top* indicates amino acids deleted in Δ584 to 604. *B,* schematic and summary of the results for Δ584 to 604. The *black bars* indicate the seven conserved nuclease motifs present in alkaline nucleases. The *N-terminal circles* represent the tag added for purification. For experiments in mammalian cells, UL12 was expressed with an N-terminal FLAG, and for experiments in bacteria, UL12 was expressed with an N-terminal GST. The figure is drawn to scale. *C,* UL12 Δ584 to 604 was expressed in 293T cells, immunoprecipitated with FLAG beads, and immunoblotted for USP15, Rad50, and FLAG as indicated. *D,* UL12 Δ584 to 604 was expressed in Arctic Express bacteria and purified on glutathione sepharose resin. After extensive washing, UL12-bound resin was incubated in 293T nuclear extracts. UL12 was visualized by Coomassie blue staining of the gel. USP15 copurifying with UL12 was identified by immunoblot. *Arrows* indicate the respective sizes of GST and UL12. GST, glutathione-*S*-transferase; HSV, herpes simplex virus; USP15, ubiquitin-specific protease 15.

To better understand the amino acids in the C-terminal alpha helix of UL12 that could contribute to USP15 binding, we performed a sequence alignment of the C-terminal helix of UL12 from human and primate simplexviruses. We observed greater than 85% amino acid sequence identity between residues 584 and 604 (Fig. 2*A*). We hypothesized that this represented the USP15-interacting domain and generated UL12 Δ584 to 604 (Fig. 2*B*). FLAG-tagged UL12 Δ584 to 604 did not interact with USP15 in coimmunoprecipitation experiments from mammalian cells but did maintain the interaction with the MRN complex (Fig. 2*C*). Again, we observed lower expression levels of this protein, presumably because it can no longer be stabilized by USP15. To control for this, we expressed UL12 Δ584 to 604 in the bacterial system. Like in mammalian cells, GST-UL12 Δ584 to 604 did not interact with USP15 (Fig. 2*D*). Finally, UL12 Δ584 to 604 was expressed at higher levels than UL12, indicating the observation is not from purifying less of the mutant protein.

**Figure 3:**
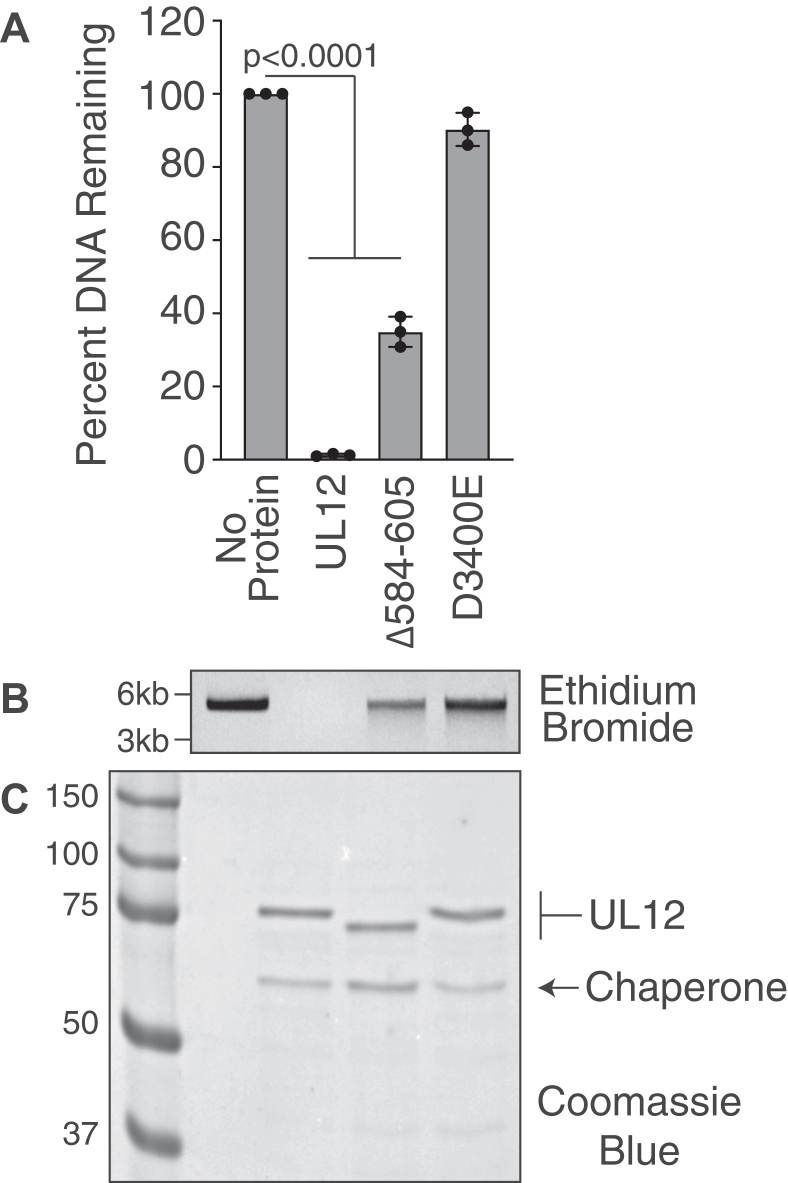
**UL12 Δ584 to 604 retains exonuclease activity.***A,* UL12, Δ584 to 604, and exonuclease-deficient D340E were purified from Arctic Express bacteria, and the GST was cleaved off by PreScission protease treatment. *A* and *B,* purified proteins were incubated with linearized plasmid DNA for 1 h. Remaining DNA was visualized by agarose gel electrophoresis and quantified as described in the Experimental procedures section. *A,* quantification of three biological replicates. The graph shows mean ± SD and ANOVA with Dunnett's post-test. *B,* a representative ethidium bromide–stained gel is shown for each protein. The larger band represents linearized plasmid DNA, and the smaller smears represent intermediate degradation products. *C,* purified proteins were visualized by Coomassie blue. *Arrows* indicate the respective sizes of UL12 and the copurifying chaperone protein. GST, glutathione-S-transferase.

UL12 Δ584 to 604 removes a portion of the C-terminal helix adjacent to the final nuclease motif of UL12. To control for possible global changes in UL12 structure, we tested the exonuclease activity of this mutant. GST-fusions of UL12, Δ584 to 604, and D340E were induced as in previous experiments and captured on glutathione sepharose resin. Proteins were released from the resin by cleavage with PreScission protease, which cuts C terminal to the GST fusion, releasing full-length UL12 with two additional amino acids at the N terminus. Purified proteins were incubated with linearized plasmid DNA for 1 h. Remaining DNA was visualized by ethidium bromide staining of agarose gels, and protein purity was assessed by Coomassie blue staining (Fig. 3, *A*–*C*). All purifications were greatly enriched for UL12 and contained the copurifying chaperone from the bacterial induction system, which we have noted in the past (38).

All DNA was completely degraded by UL12, and there was little to no degradation with the exonuclease-deficient D340E protein (Fig. 3, *A* and *B*), consistent with the published literature (27). The lack of exonuclease activity with D340E indicates that the activity is specific to UL12 and not the copurifying chaperone protein. We observed that Δ584 to 604 maintained about 60% to 70% of the exonuclease activity compared with UL12. Since Δ584 to 604 maintains the protein–protein interaction with MRN and some exonuclease activity, we conclude that the protein is not globally misfolded.

### USP15 interacts with alphaherpesvirus but not other human betaherpesvirus or gammaherpesvirus UL12 homologs

**Figure 4:**
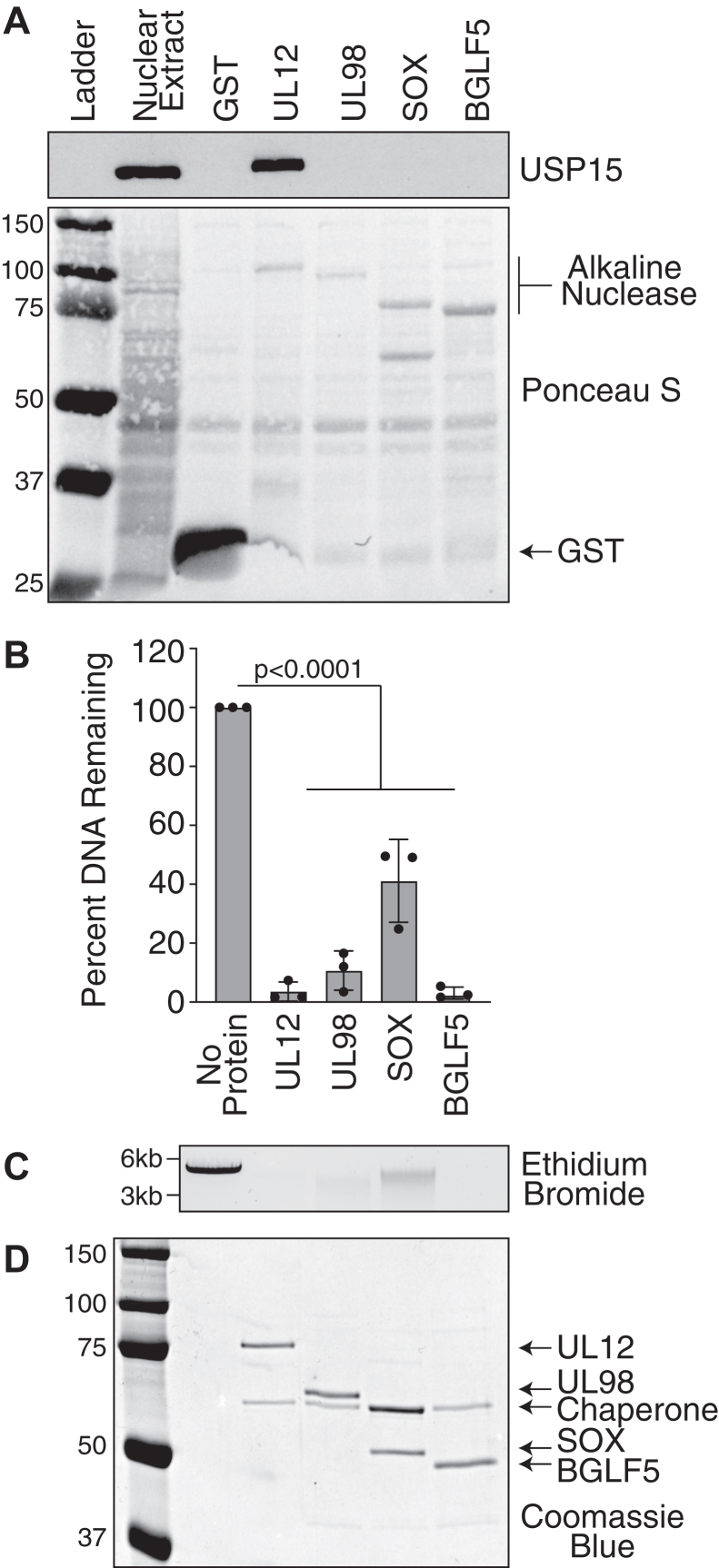
**Human betaherpesvirus and gammaherpesvirus alkaline nucleases do not interact with USP15.***A,* the indicated beta (HCMV UL98) and gamma (KSHV SOX and EBV BGLF5) alkaline nucleases were expressed in Arctic Express bacteria and purified on glutathione sepharose resin. After extensive washing, alkaline nuclease–bound resin was incubated in 293T nuclear extracts. Alkaline nucleases were visualized by Ponceau S staining of the membrane. *Arrows* indicate the respective sizes of GST and alkaline nucleases. USP15 copurifying with alkaline nuclease was identified by immunoblot. *B*–*D,* proteins were purified as described above, and the GST was cleaved off by PreScission protease treatment. Purified proteins were incubated with linearized plasmid DNA for 1 h. Remaining DNA was visualized by agarose gel electrophoresis and quantified as described in the Experimental procedures section. *B,* quantification of three biological replicates. The graph shows mean ± SD and ANOVA with Dunnett's post-test. *C,* a representative ethidium bromide–stained gel is shown. The larger band represents linearized plasmid DNA, and the smaller smears represent intermediate degradation products. *D,* purified proteins were visualized by Coomassie blue. *Arrows* indicate the respective sizes of different alkaline nucleases and the copurifying chaperone protein. EBV, Epstein–Barr virus; GST, glutathione-*S*-transferase; HCMV, human cytomegalovirus; KSHV, Kaposi's sarcoma–associated herpesvirus; USP15, ubiquitin-specific protease 15.

Since the USP15-interacting domain on UL12 was so highly conserved in other simplexviruses, we determined whether other human herpesvirus alkaline nuclease homologs from one betaherpesvirus and both gammaherpesviruses can also interact with USP15. GST, GST-UL12, GST-UL98 (beta), GST-SOX (gamma), and GST-BGLF5 (gamma) were induced at low temperatures, and soluble lysates were incubated with glutathione sepharose resin. Protein-bound resin was then incubated with 293T nuclear extracts, and copurifying USP15 was identified by immunoblotting. USP15 was efficiently pulled down with UL12 but not with UL98, SOX, BGLF5, or GST alone (Fig. 4*A*).

To verify that UL98, SOX, and BGLF5 were folded correctly, we tested their exonuclease activity. GST-fusion proteins were induced as in previous experiments and captured on glutathione sepharose resin. Proteins were released from the resin by cleavage with PreScission protease and incubated with linearized plasmid DNA for 1 h. Remaining DNA was visualized by ethidium bromide staining of agarose gels, and protein purity was assessed by Coomassie blue staining (Fig. 4, *B*–*D*). All purifications were greatly enriched for the protein of interest and contained the copurifying bacterial chaperone. All three proteins exhibited exonuclease activity, indicating they are likely folded correctly.

### The structure of the USP15-interacting domain is conserved in alphaherpesviruses but not gammaherpesviruses

**Figure 5:**
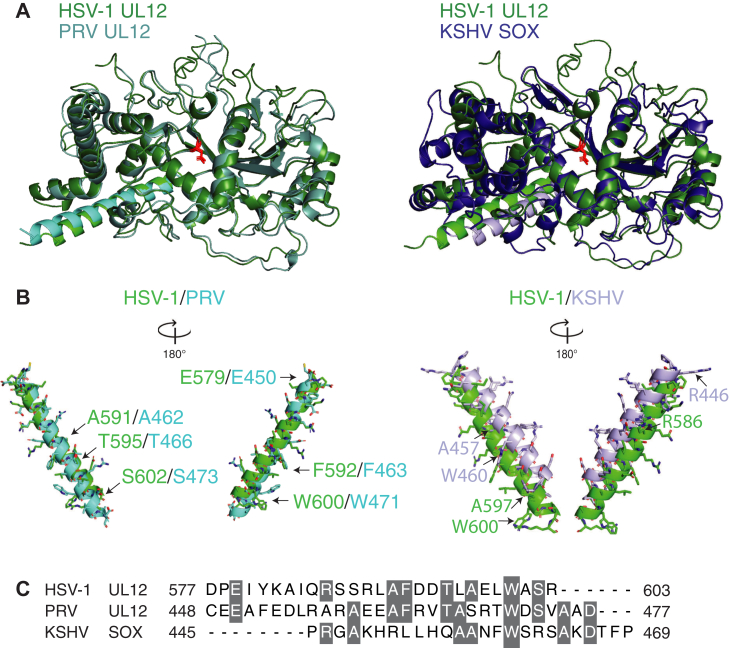
**HSV-1 UL12 C-terminal helix has structural and sequence homology with other alphaherpesvirus but not gammaherpesvirus alkaline nucleases.***A,* structure overlay of HSV-1 UL12 (*green*), alphaherpes PRV UL12 (*blue*), and gammaherpes KSHV SOX (*purple*). The C-terminal helices are highlighted in *light green*, *light blue*, and *light purple*, respectively. The central aspartic acid highlighted in *red* shows the location of the exonuclease domain. *B,* overlay of the HSV-1 UL12 helix with PRV UL12 and KSHV SOX. Identical amino acids are indicated with *arrows*. All helices are oriented with the N-terminal amino acids at the *top* of the figure and C-terminal amino acids at the *bottom* of the figure. *C,* amino acid sequence alignment of the C-terminal helix from each virus. Identical amino acids are highlighted in *gray*. HSV, herpes simplex virus; KSHV, Kaposi's sarcoma–associated herpesvirus; PRV, pseudorabies virus.

To better understand the USP15-interacting domain at a structural level, we took advantage of HerpesFolds, a new structural prediction tool for all human herpesviruses and some veterinary herpesviruses (37). HerpesFolds predicted a consistent structure for all nine human herpesvirus alkaline nucleases and demonstrated that they contain a structured core with N- and C-terminal extensions. The predicted structures were also consistent with the published crystal structures of EBV BGLF5 and KSHV SOX (37, 39, 40, 41). We compared the structures of HSV-1 UL12 with pseudorabies virus (PRV) UL12, another alphaherpesvirus, and with KSHV SOX, a gammaherpesvirus. In general, we observed a strong structural similarity between the three proteins and observed that the essential HSV-1 UL12 catalytic residue D340, highlighted in red, is in the same place in all structures (Fig. 5*A*).

Although the proteins are largely folded in a similar manner, we focused on the C-terminal alpha helix that we propose is the USP15-interacting domain. PRV is a varicellovirus, and while still an alphaherpesvirus, its UL12 protein is less conserved with HSV-1 than the other simplexviruses analyzed, which have very high sequence identity in the USP15-interacting domain (Fig. 2*A*). We observed that the helices from HSV-1 and PRV UL12 overlap very well in the predicted structures. They also contain six identical residues in the USP15-interacting domain in identical positions in the helix (Fig. 5, *B left structures* and *C*). KSHV SOX is the gammaherpesvirus nuclease, and the C-terminal helix is shorter than that observed in UL12 and does not overlap well in the predicted structures. Sequence alignments highlight a few conserved amino acids between UL12 and SOX; however, these residues do not map near each other in the aligned structures, suggesting they may not be functionally redundant as in PRV UL12 (Fig. 5, *B right structures* and *C*).

### UL12 C-terminal helices in alphaherpesviruses represent a functional USP15-interacting domain that can be swapped between UL12 homologs

**Figure 6:**
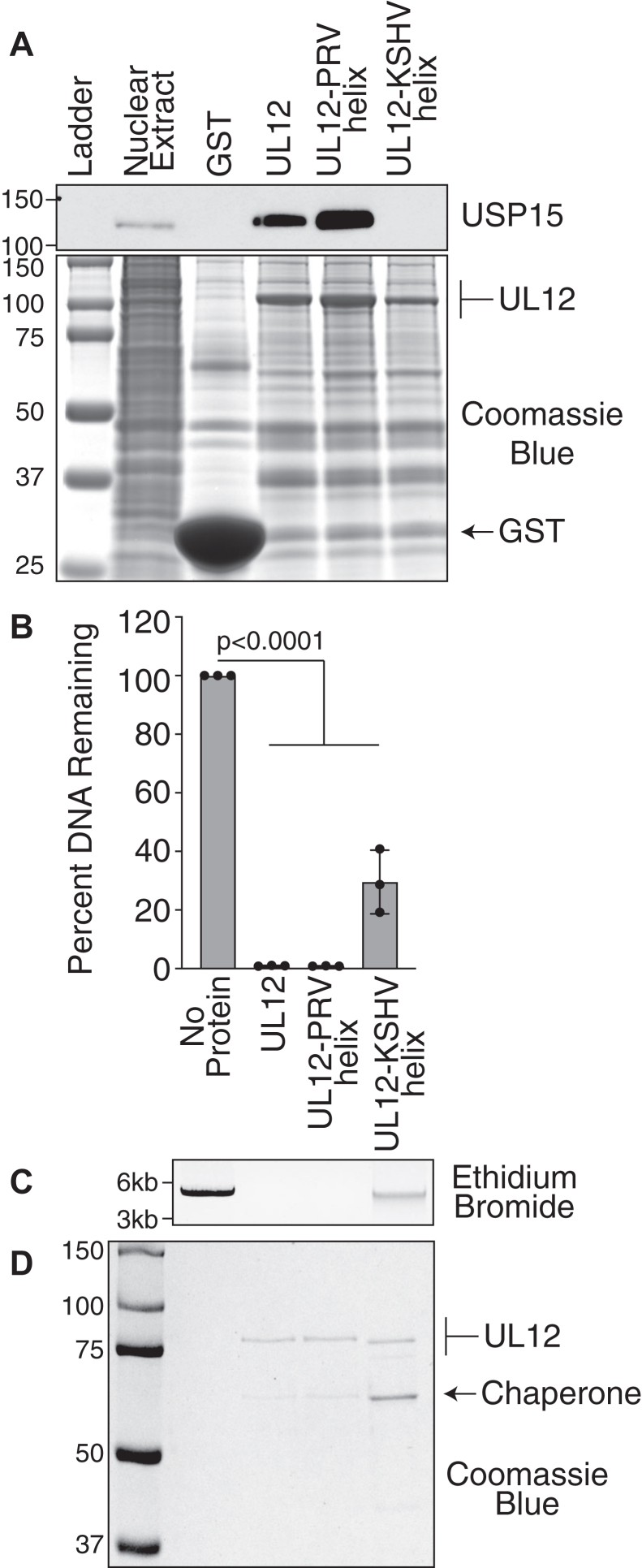
**Alphaherpes but not gammaherpes alkaline nuclease C-terminal helix is a USP15-interacting domain.***A,* the indicated alpha (PRV UL12) and gamma (KSHV SOX) C-terminal helices were cloned in place of the UL12 C-terminal helix. Proteins were expressed in Arctic Express bacteria and purified on glutathione sepharose resin. After extensive washing, UL12-bound resin was incubated in 293T nuclear extracts. UL12 was visualized by Coomassie blue staining of the gel. *Arrows* indicate the respective sizes of GST and UL12. USP15 copurifying with UL12 was identified by immunoblot. *B*–*D,* proteins were purified as described above, and the GST was cleaved off by PreScission protease treatment. Purified proteins were incubated with linearized plasmid DNA for 1 h. Remaining DNA was visualized by agarose gel electrophoresis and quantified as described in the Experimental procedures section. *B,* quantification of three biological replicates. The graph shows mean ± SD and ANOVA with Dunnett's post-test. *C,* a representative ethidium bromide–stained gel is shown. The larger band represents linearized plasmid DNA, and the smaller smears represent intermediate degradation products. *D,* purified proteins were visualized by Coomassie blue. *Arrows* indicate the respective sizes of UL12 and the copurifying chaperone protein. GST, glutathione-*S*-transferase; KSHV, Kaposi's sarcoma–associated herpesvirus; PRV, pseudorabies virus; USP15, ubiquitin-specific protease 15.

We propose that the UL12 C-terminal helix represents a *bona fide* USP15 interacting module conserved in alphaherpesviruses. To test this hypothesis, we swapped the C-terminal helix of UL12 with the corresponding sequences from PRV UL12 and KSHV SOX. Helix swap mutants GST-UL12-PRV helix (HSV UL12 with the C-terminal helix from PRV UL12) and GST-UL12-KSHV helix (HSV UL12 with the C-terminal helix from KSHV SOX) were induced at low temperatures, and soluble lysates were incubated with glutathione sepharose resin. Protein-bound resin was then incubated with 293T nuclear extracts, and copurifying USP15 was identified by immunoblotting. USP15 was efficiently pulled down with UL12 and UL12-PRV helix but not with UL12-KSHV helix or GST alone (Fig. 6*A*). Of note, UL12-PRVhelix seems to bind USP15 better than wildtype UL12, perhaps indicating that some of the amino acids in the PRV helix may increase binding affinity. These data demonstrate that the UL12 C-terminal alphaherpesvirus helix is necessary for USP15 interaction.

To ensure that UL12-PRV helix and UL12-KSHV helix are folded correctly, we determined if the proteins still retained exonuclease activity as previously described. Both proteins exhibited exonuclease activity, indicating that they are likely folded correctly (Fig. 6, *B* and *C*). UL12-KSHV helix had slightly less activity in this experiment, consistent with the slightly lower activity of SOX observed in Figure 4*B*, suggesting that this is not unique to the hybrid protein. All purifications were greatly enriched for the protein of interest and contained the copurifying bacterial chaperone (Fig. 6*D*). UL12-KSHV helix is slightly smaller than the other proteins because of the slightly shorter helix present in SOX (Figs. 5*C* and 6*D*). We also observed more chaperone proteins copurifying with UL12-KSHV helix than with the other proteins. This is consistent with the increased amount of chaperone purified with KSHV Sox alone and the slightly reduced exonuclease activity compared with the other homologs (Fig. 4, *B*–*D*).

**Figure 7:**
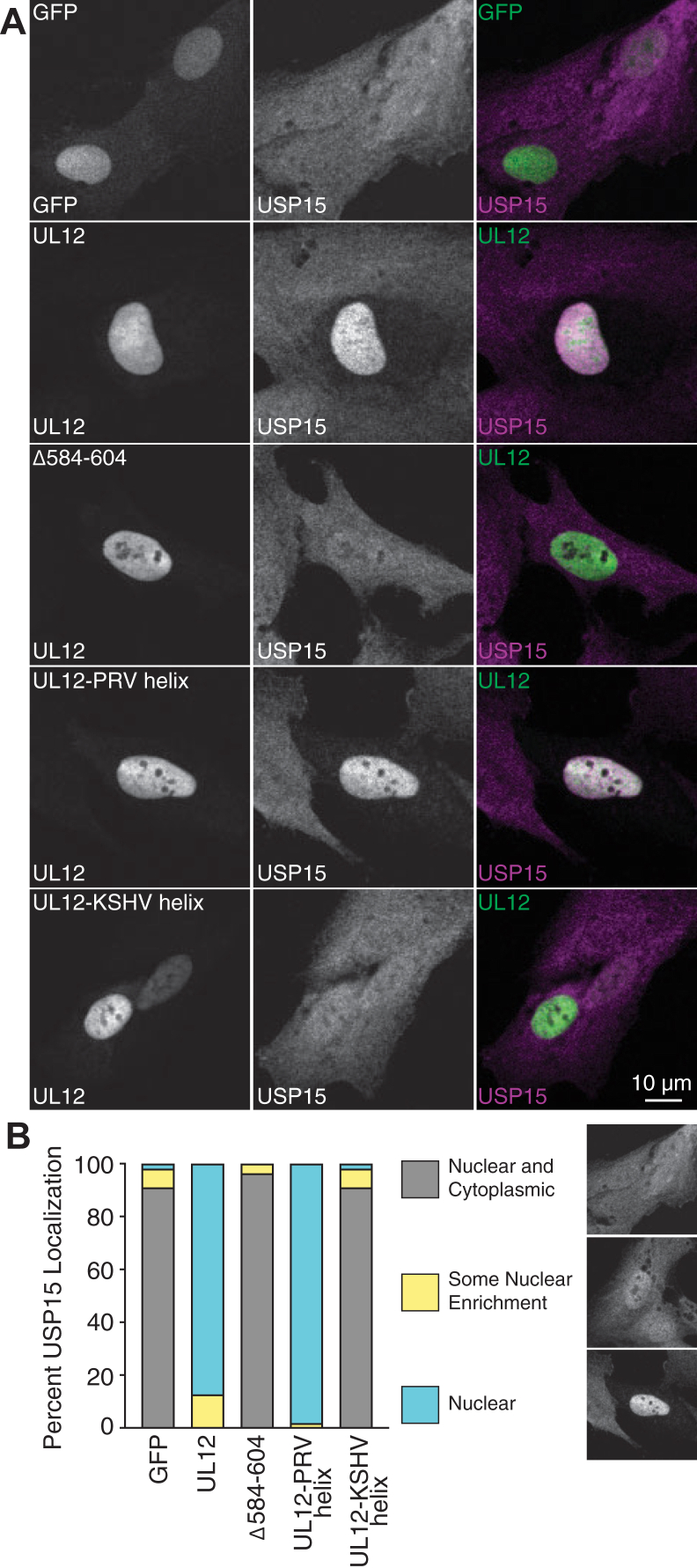
**USP15-interacting C-terminal helices are functional and support UL12-mediated nuclear reorganization of USP15.** RPE–hTERT cells were transfected with GFP expressing a nuclear localization signal or the indicated FLAG-UL12 deletions and mutants. Cells were fixed at 24 h post-transfection and stained with FLAG and USP15 antibodies. *A,* representative images showing UL12 and UL12-PRV helix can recruit USP15 to the nucleus. The scale bar represents 10 μm. *B,* quantification of USP15 localization. At least 50 transfected cells were analyzed per condition. Pictures represent an example of each classification used. hTERT, human telomerase reverse transcriptase; PRV, pseudorabies virus; RPE, retinal pigment epithelium; USP15, ubiquitin-specific protease 15.

We have previously published that UL12 expression is sufficient to reorganize USP15 from a cellular diffuse staining pattern into the nucleus in transfection-based experiments (30). To further test our hypothesis that the UL12 C-terminal alphaherpesvirus helix is a USP15-interacting domain in cells, we tested the ability of UL12-PRV helix and UL12-KSHV helix to alter the cellular localization of USP15. Following transfection of wildtype or mutant UL12, we observed that proteins that can interact with USP15, UL12, and UL12-PRV helix were sufficient to reorganize USP15 to the nucleus. Conversely, proteins that cannot interact with USP15, Δ584 to 604, and UL12-KSHV helix were not sufficient to reorganize USP15 to the nucleus (Fig. 7, *A* and *B*). Consistent with the slightly increased binding of UL12-PRV helix to USP15, we also observed that this protein recruited USP15 to the nucleus a little more efficiently than wildtype UL12. Together, these data indicate that the UL12 C-terminal alphaherpesvirus helix is necessary for USP15 interaction and UL12-dependent localization of USP15 in cells, indicating a functional USP15-interacting domain.

### Conserved amino acids between HSV-1 UL12 and PRV UL12 in the C-terminal helix are required for the interaction with USP15

**Figure 8:**
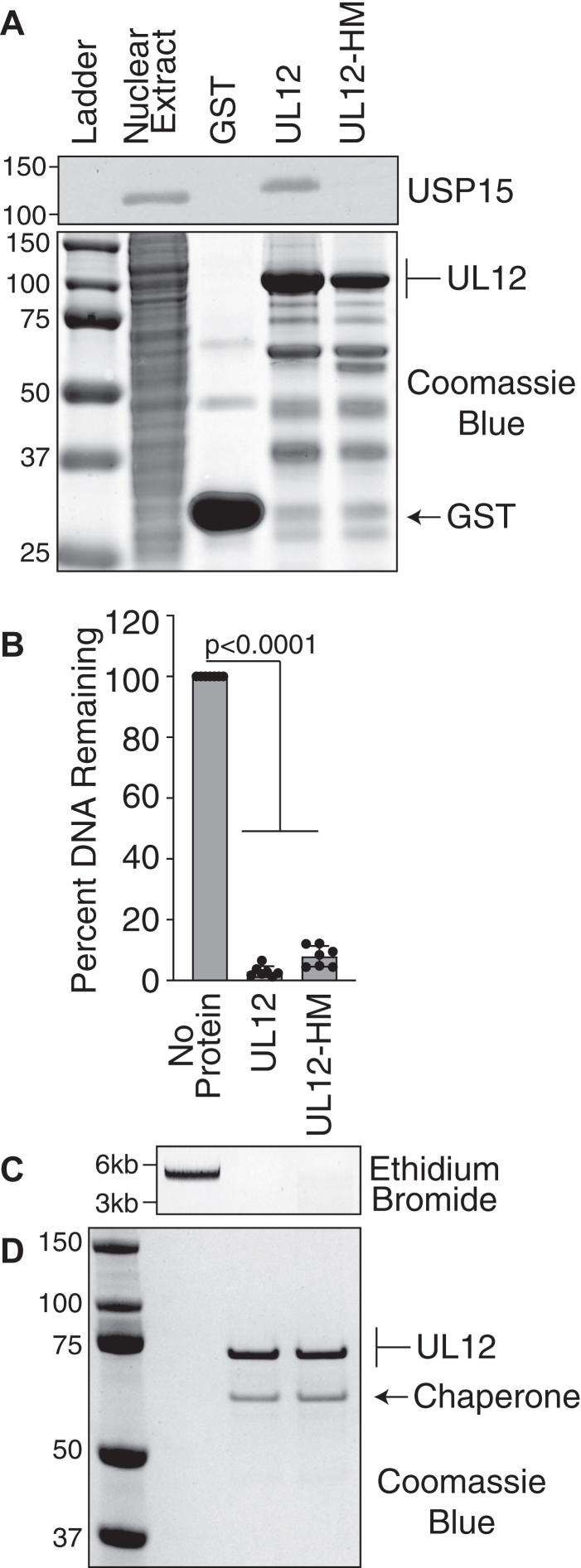
**Conserved amino acids between HSV-1 UL12 and PRV UL12 in the C-terminal helix are required for the interaction with USP15.***A,* the indicated UL12 and UL12-HM (helix mutant) proteins were expressed in Arctic Express bacteria and purified on glutathione sepharose resin. After extensive washing, UL12-bound resin was incubated in 293T nuclear extracts. UL12 was visualized by Coomassie blue staining of the gel. *Arrows* indicate the respective sizes of GST and UL12. USP15 copurifying with UL12 was identified by immunoblot. *B*–*D,* proteins were purified as described above, and the GST was cleaved off by PreScission protease treatment. Purified proteins were incubated with linearized plasmid DNA for 1 h. Remaining DNA was visualized by agarose gel electrophoresis and quantified as described in the Experimental procedures section. *B,* quantification of seven biological replicates. The graph shows mean ± SD and ANOVA with Dunnett's post-test. *C,* a representative ethidium bromide–stained gel is shown. The larger band represents linearized plasmid DNA, and the smaller smears represent intermediate degradation products. *D,* purified proteins were visualized by Coomassie blue. *Arrows* indicate the respective sizes of UL12 and the copurifying chaperone protein. GST, glutathione-*S*-transferase; HSV, herpes simplex virus; PRV, pseudorabies virus.

The C-terminal helix of HSV-1 UL12 and PRV UL12 contains similar protein folds as well as six identical amino acids (Fig. 5, *B* and *C*). To further define whether structural similarity or amino acid identity governs the interaction between the UL12 C-terminal helix and USP15, we mutated three of the six identical amino acids to generate HSV-1 UL12-Helix Mutant (UL12-HM). UL12-HM contains A591G/F592A/W600A. We excluded mutating the conserved serine and threonine, as UL12 is known to be heavily phosphorylated, and there is evidence of phosphorylation in this region (42). GST-UL12 and GST-UL12-HM were induced at low temperatures, and soluble lysates were incubated with glutathione sepharose resin. Protein-bound resin was then incubated with 293T nuclear extracts, and copurifying USP15 was identified by immunoblotting. USP15 was efficiently pulled down with UL12 but not with UL12-HM or GST alone (Fig. 8*A*). These data demonstrate that some of the identical UL12 C-terminal amino acids in HSV-1 UL12 and PRV UL12 are necessary for the USP15 interaction.

To ensure that UL12-HM is folded correctly, we determined if the protein still retained exonuclease activity as previously described. Both UL12 and UL12-HM proteins exhibited similar exonuclease activity (Fig. 8, *B* and *C*). Of note, UL12-HM retains considerably more exonuclease activity than the helix deletion mutant Δ584 to 604, indicating that it is likely folded correctly. All purifications were greatly enriched for the protein of interest and contained the copurifying bacterial chaperone (Fig. 8*D*).

**Figure 9:**
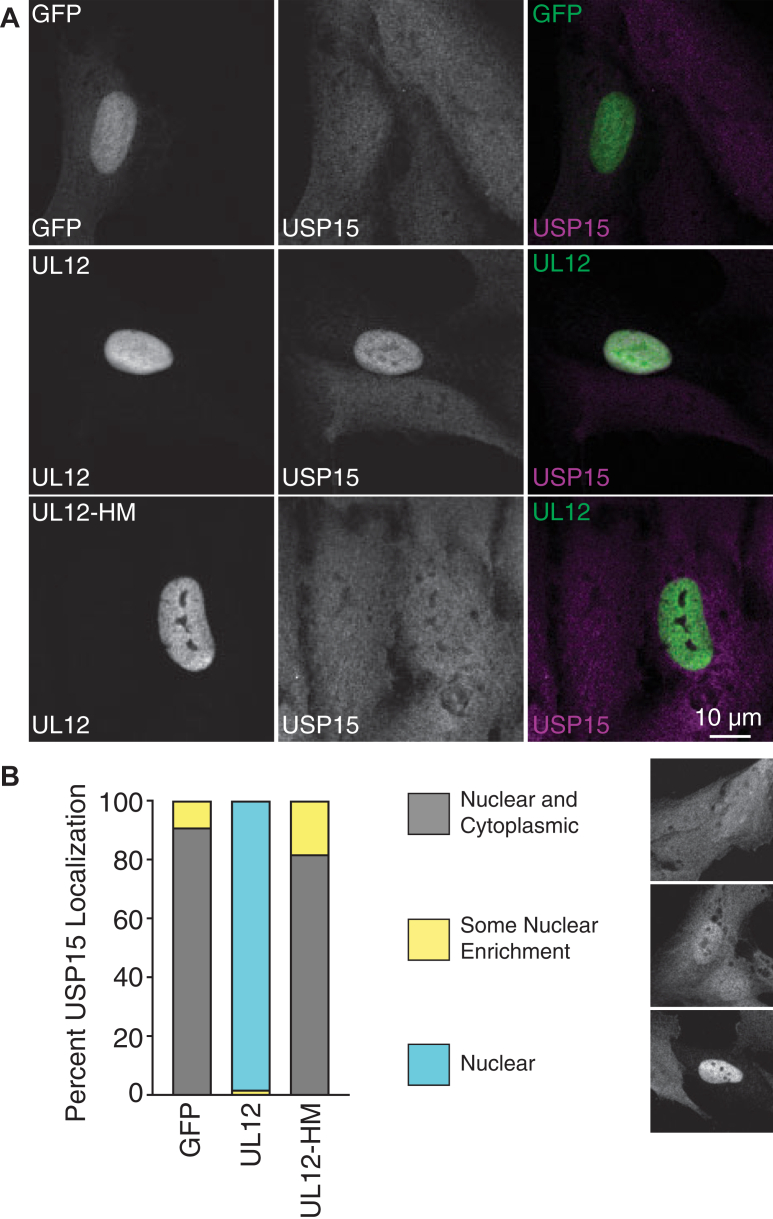
**Conserved amino acids between HSV-1 UL12 and PRV UL12 in the C-terminal helix are required for UL12-mediated nuclear reorganization of USP15.** RPE–hTERT cells were transfected with GFP expressing a nuclear localization signal, FLAG-UL12, or FLAG-UL12-HM (helix mutant). Cells were fixed at 24 h post-transfection and stained with FLAG and USP15 antibodies. *A,* representative images showing UL12 but not UL12-HM can recruit USP15 to the nucleus. The scale bar represents 10 μm. *B,* quantification of USP15 localization. At least 50 transfected cells were analyzed per condition. Pictures represent an example of each classification used, as also indicated in Figure 7. HSV, herpes simplex virus; hTERT, human telomerase reverse transcriptase; PRV, pseudorabies virus; RPE, retinal pigment epithelium.

To further test our hypothesis that the identical amino acids in the UL12 C-terminal helix are necessary for USP15 interaction, we tested the ability of HSV-1 UL12-HM to recruit USP15 to the nucleus in transfected cells. UL12 was able to recruit USP15 to the nucleus in all transfected cells observed. UL12-HM was not able to recruit USP15 to the nucleus and showed similar USP15 localization as the GFP transfection control (Fig. 9, *A* and *B*). Together, these data indicate that the identical UL12 C-terminal amino acids are necessary for USP15 interaction and UL12-dependent localization of USP15 in cells, indicating critical amino acids in the USP15-interacting domain.

## Discussion

In this study, we identified the C-terminal alpha helix of UL12 to be a USP15-interacting domain and functionally validated three key amino acids required for the interaction. This domain was necessary for UL12 to bind USP15 in both mammalian and bacterial expression systems. Furthermore, point mutations generated in this domain did not significantly alter UL12 exonuclease activity, indicating that the mutant protein is likely folded correctly. This USP15-interacting domain was also essential for UL12 to recruit USP15 to the nucleus. We observed no binding to HCMV UL98, KSHV SOX, or EBV BGLF5 proteins. We were also able to demonstrate that the helix is a functional domain by swapping the HSV-1 UL12 helix with the corresponding PRV UL12 helix and still maintain the interaction with USP15. Conversely, the KSHV SOX helix was not able to support USP15 binding when added in place of the HSV-1 UL12 helix. In addition to supporting USP15 binding, the PRV UL12 helix also supported recruitment of USP15 to the nucleus, whereas the KSHV SOX helix could not. Analysis of the sequence identity of HSV and PRV helices indicated the presence of six identical amino acids. Mutation of three of these amino acids on HSV-1 UL12 was sufficient to disrupt the interaction in both pull-down and cell-based USP15 interaction assays, thus functionally validating essential amino acids in the USP15-interacting domain.

The UL12 C-terminal helix is preserved in all human herpesviruses but only retains the ability to bind USP15 in alphaherpesviruses. Sequence alignments of the UL12 helix from other alphaherpesviruses show over 80% to 85% sequence identity between the proteins. This core of conserved amino acids represents the USP15-interacting domain, as deletion results in the inability of UL12 to bind USP15 or recruit it to the nucleus. We expanded our study to include the additional alphaherpesvirus UL12 from PRV, which has a slightly more diverged amino acid composition than HSV-1 UL12. This PRV UL12 helix shows excellent structural alignment with HSV-1 UL12 and still maintains the binding to USP15 and recruitment of USP15 to the nucleus. The KSHV SOX protein also has a C-terminal helix in the same position, but the sequence identity is highly divergent, and the helix structures do not align well. This indicates that while the C-terminal helix is present in all herpesviruses, it is only a USP15-interacting domain in alphaherpesviruses. These data indicate how the regulation of conserved enzymes in a protein or a virus family can diverge throughout evolution. While all known human herpesviruses contain an alkaline nuclease, the regulation of this enzyme has clearly diverged in different virus lineages. This could also be a feature of how long each of these viruses has been coevolving with humans. HSV-1 was introduced into human ancestors about 6 million years ago and has been coevolving ever since (43). Conversely, the earliest common human KSHV ancestor has only entered the human population about 100,000 years ago (44).

Several studies have looked at the global protein interactions of other herpesviruses, and none have identified USP15 as an interactor of the respective alkaline nuclease. A study of HCMV protein interactions utilized a C-terminal V5 tag on UL98 and identified only one high-confidence interacting protein in human foreskin fibroblast–telomerase reverse transcriptase (TERT) cells, serrate RNA effector molecule (SRRT) (45). Another study utilized C-terminal 2X strep-tagged SOX protein expressed in 293T cells and identified eight high-confidence interacting proteins. These proteins were largely involved in RNA metabolism (ADAR1, RNase P subunit p40, RNase P subunit p30, TRNA Methyltransferase 61, and PUS7) and ISWI chromatin remodeling complex protein BAZ1A (46). Finally, C-terminal hemagglutinin-tagged BGLF5 in the P3HR1 Burkitt lymphoma cell line had eight high-confidence interacting proteins with roles in RNA metabolism (TAF4, DDX1, and RNase P subunit p20), guanidine nucleotide exchange (ARHGEF1), and protein phosphatases (Liprin-alpha-1 and striatin) (47). While none of these studies explicitly validated the interacting proteins with alkaline nuclease, the protein interactions with SOX and BGLF5 are consistent with their additional function of host shutoff, in which they accelerate the degradation of messenger RNA during infection (48, 49).

This study extends our understanding about the regulation of HSV-1 UL12 stability through interacting with cellular USP15. It also highlights an evolutionarily conserved USP15 interaction domain in the C-terminal helix of alphaherpesvirus alkaline nucleases. While every herpesvirus includes an alkaline nuclease, the regulation of these proteins appears to have diverged in the betaherpesviruses and gammaherpesviruses.

## Experimental procedures

### Cell lines

293T and retinal pigment epithelium (RPE)–human TERT (hTERT) cells were obtained from the American Type Culture Collection and routinely tested for mycoplasma. 293T cells were cultured in Dulbecco's modified Eagle's medium supplemented with 7.5% fetal bovine serum. RPE–hTERT cells were cultured in Dulbecco's modified Eagle's medium/F12 supplemented with 7.5% fetal bovine serum and 0.25% sodium bicarbonate.

### Plasmids

Plasmids pLEGFP-FLAG-NLS (pDC1161), pLEGFP-FLAG-NLS UL12 (pKM506), pLPG-FLAG (pTB114), pLPG-FLAG-UL12 (pKM507), pSAK-UL12-D340E, and His-GST-3C (pBG101) were previously described (10, 30). Truncations of the HSV1-UL12 gene were constructed by cloning PCR-amplified fragments of UL12 into pDC1161 to generate amino acids 2 to 126 (pAL9), 2 to 271 (pAL14), 2 to 490 (pAL17), 2 to 577 (pAL22), 2 to 583 (pAL26), 2 to 606 (pAL23), and 128 to 626 (pAL10). UL12 was also cloned into pBG101 to generate His-GST-3C-UL12 (pKM540), 2 to 577 (pAL95), and 2 to 606 (pAL97). Mutagenesis PCR was conducted on pKM540 to delete amino acids 584 to 604 and make His-GST-3C-UL12Δ584 to 604 (pAL102). UL12Δ584 to 604 was subcloned into pTB114 to make pLPG-FLAG-UL12Δ584 to 604 (pAL30). HCMV UL98 was amplified from AD169-BAC4 (50) and cloned into pBG101 (pAL80). KSHV SOX was amplified from pcDNA4-TO-ORF37-2xCSTREP, a gift from Dr Britt Glaunsinger (Addgene plasmid #136198) (46), and cloned into pBG101 (pAL81). EBV BGLF5 was amplified from pENTR223-BGLF5, a gift from Eric Johannsen (Addgene plasmid #195813) (51) and cloned into pBG101 (pAL78). UL12 D340E was PCR amplified from pSAK-UL12-D340E and cloned into pBG101 (pKM541). UL12 with amino acids 577 to 603 replaced with either amino acids 448 to 477 from PRV UL12 (UL12-PRV helix) or amino acids 445 to 469 from KSHV SOX (UL12-KSHV helix) were synthesized by Azenta Bioscience and cloned into pBG101 to generate pAL85 and pAL86, respectively. UL12-HM (A591G/F592A/W600A) was made by site-directed mutagenesis of pKM540 at Azenta Bioscience to generate pAL65. UL12, Δ584 to 604, UL12-PRV helix, UL12-KSHV helix, and UL12-HM were also cloned into pLPCX-FLAG-hemagglutinin (pKM331) to generate pAL38, pAL75, pTDH36, pTDH37, and pAL110, respectively.

### Nuclear extracts and coimmunoprecipitation

Nuclear extracts were prepared by the method of Dignam from 293T cells transfected with FLAG-tagged proteins (52). Briefly, cells were scraped into PBS and pelleted; the PBS was discarded, and the pellet was rapidly resuspended in five packed cell volumes of hypotonic buffer and pelleted again. The cell pellet was then resuspended in hypotonic buffer to a final volume of three packed cell volumes and allowed to swell on ice for 10 min. The cells were transferred to a glass Dounce homogenizer and homogenized with 10 to 20 up-and-down strokes with a type B pestle. The nuclei were collected by centrifuging for 15 min at 3300*g*. The packed nuclear volume was estimated, and the nuclei were resuspended in a volume of low-salt buffer equal to half the packed nuclear volume. An equal volume of high-salt buffer was added in a dropwise fashion with constant stirring. The nuclei were supplemented with 250 U/ml Pierce Universal Nuclease and allowed to extract for 30 min at 4 °C with constant gentle mixing. The extracted nuclei were pelleted for 30 min at 12,000*g*. The resulting supernatant was dialyzed against 50 volumes of dialysis buffer for 30 to 60 min. After dialysis, soluble nuclear proteins were immunoprecipitated overnight at 4 °C using EZview Red FLAG M2 affinity gel. The affinity gel was then washed three times with dialysis buffer, once with elution buffer, and then interacting proteins were eluted with elution buffer supplemented with 0.3 mg/ml FLAG peptide on ice for 1 h. Samples were then analyzed by immunoblotting. Hypotonic buffer is 10 mM Hepes (pH 7.9) at 4 °C, 1.5 mM MgCl_2_, and 10 mM KCl. Low-salt buffer is 20 mM Hepes (pH 7.9) at 4 °C, 25% glycerol, 1.5 mM MgCl_2_, and 20 mM KCl. High-salt buffer is 20 mM Hepes (pH 7.9) at 4 °C, 25% glycerol, 1.5 mM MgCl_2_, and 1.2 M KCl. Dialysis buffer is 20 mM Hepes (pH 7.9) at 4 °C, 20% glycerol, 1.5 mM MgCl_2_, and 100 mM KCl. FLAG elution buffer is 10 mM Hepes (pH 7.9) at 4 °C, 1.5 mM MgCl_2_, 300 mM KCl, and 0.05% NP-40. All buffers were freshly supplemented with 5 μg/ml aprotinin, 5 μg/ml leupeptin, and 1 mM DTT, except dialysis buffer, which was supplemented with 0.2 mM PMSF and 0.5 mM DTT immediately prior to use.

### Protein purification

GST fusion proteins were induced with 1 mM IPTG overnight at 13 °C in Arctic Express (DE3) *Escherichia coli* (Agilent Technologies). Cell pellets were resuspended in NET buffer (25 mM Tris [pH 8.0], 50 mM NaCl, 0.1 mM EDTA, 5% glycerol, 1 mM DTT, 0.1 mM PMSF, 5 μg/ml aprotinin, and 5 μg/ml leupeptin) and lysed by sonication. Triton X-100 was added to a final concentration of 1%, and the lysate was incubated on ice for 30 min. Following high-speed centrifugation, the lysate was incubated with Glutathione Sepharose 4B resin (Cytiva) at 4 °C overnight.

To analyze protein–protein interactions, the protein-bound resin was washed three times with NET buffer containing 1% Triton X-100 and once with dialysis buffer (20 mM Hepes [pH 7.9] at 4 °C, 20% glycerol, 100 mM KCl, 1.5 mM MgCl_2_, 0.2 mM PMSF, and 0.5 mM DTT). The resin was then incubated with 293T nuclear extracts at 4 °C overnight. The resin was then washed three times with dialysis buffer, and proteins were eluted by boiling in 2X SDS sample buffer.

To generate protein for exonuclease assays, the protein-bound resin was washed three times with NET buffer containing 1% Triton X-100, twice with protease cleavage buffer (25 mM Tris [pH 8.0], 50 mM NaCl, 0.1 mM EDTA, and 1 mM DTT), and then resuspended in protease cleavage buffer with PreScission Protease (GenScript) at 4 °C overnight. The resin was then centrifuged, and the supernatant containing the purified protein was collected. Glycerol was added to a final concentration of 5%, and proteins were stored at −80 °C.

### Exonuclease assay

Exonuclease assays were performed as previously described (27). Briefly, 300 ng of protein was added to 100 ng of NotI-linearized pcDNA3 in a 20 μl reaction volume with 50 mM Tris (pH 8.8), 10 mM MgCl_2_, and 5 mM DTT. The reactions were incubated at 37 °C for 1 h and stopped by the addition of 6X gel loading dye containing SDS and EDTA (New England Biolabs). The products were subjected to electrophoresis on a 0.7% agarose gel and stained with ethidium bromide. Gel images were acquired and analyzed with an iBright Imaging System (Thermo Fisher).

### Immunoblotting

Samples were mixed with 2X SDS sample buffer and boiled before being resolved by SDS-PAGE and transferred to nitrocellulose membranes. Membranes were stained with Ponceau S to visualize total proteins, destained, and blocked for 1 h with 5% nonfat dry milk dissolved in Tris-buffered saline with Tween-20. Primary antibodies were diluted in blocking solution and incubated overnight at 4 °C. Primary antibodies include mouse monoclonal anti-FLAG M2 (1:10,000 dilution; Sigma, catalog no.: F1804), rabbit monoclonal anti-USP15 D1K6S (1:10,000 dilution; Cell Signaling Technologies, catalog no.: 66310S), mouse monoclonal anti-Mre11 (1:5000 dilution; GeneTex, catalog no.: GTX70212), and mouse monoclonal anti-Rad50 (1:3000 dilution; Novus Biological, catalog no.: NB100-147). Horseradish peroxidase–conjugated secondary antibodies (Jackson ImmunoResearch) and Pierce ECL Western Blotting Substrate (Thermo Scientific) were used to develop immunoblots on HyBlot CL autoradiography film (Thomas Scientific).

### Immunofluorescence

Immunofluorescence was performed as previously described (53). Briefly, cells on glass coverslips were washed with PBS, fixed for 10 min with 4% paraformaldehyde, and then permeabilized for 2 min with ice-cold acetone. Cells were blocked with 1% bovine serum albumin in PBS and then reacted with antibodies as indicated. Primary antibodies include rabbit monoclonal anti-USP15 D1K6S (1:100 dilution; Cell Signaling Technologies, catalog no.: 66310S) and mouse monoclonal anti-FLAG M2 (1:200 dilution; Sigma, catalog no.: F1804). Alexa Fluor secondary antibodies (1:200 dilution; Invitrogen) were used with fluorophores excitable at wavelengths of 488 and 594. RPE-hTERT cells were transfected with FuGENE HD reagent (Promega) according to the manufacturer's suggested protocol. USP15 localization was scored manually.

### Structures

HerpesFolds predictions of HSV-1 UL12 (HSV-1_UL12_rank_002.pdb), PRV UL12 (PrV_UL12_rank_005.pdb), and KSHV SOX (KSHV_ORF37_rank_002.pdb) were downloaded from www.herpesfolds.org and visualized with PyMOL by overlaying PRV UL12 and KSHV SOX onto HSV UL12 (37).

### Sequence alignments

Sequence alignments for HSV-1 UL12 (YP_009137086), HSV-2 UL12 (YP_009137163), ChHV UL12 (YP_009010998), McAHV1 UL12 (NP_851871), and CeAHV2 UL12 (YP_164454) were performed with Clustal Omega and visualized with SnapGene.

## Data availability

All data described are contained within the article.

## Conflict of interest

The authors declare that they have no conflicts of interest with the contents of this article.
